# Biomanufacturing in low Earth orbit: A paradigm shift

**DOI:** 10.1016/j.stemcr.2025.102536

**Published:** 2025-06-19

**Authors:** Davide Marotta, Noor Ward, Steven R. Bauer, Joshua Hunsberger, Jana Stoudemire, Kenneth Savin, Marc Giulianotti, Catriona H.M. Jamieson, Donna Roberts, Michael Roberts

**Affiliations:** 1 International Space Station National Laboratory, Melbourne, Florida, USA; 2 Wake Forest Institute for Regenerative Medicine, Winston-Salem, North Carolina, USA; 3 Axiom Space, Houston, Texas, USA; 4 Redwire Space, Jacksonville, Florida, USA; 5 Sierra Space Corp., Louisville, Colorado, USA; 6 UC San Diego Sanford Stem Cell Institute, La Jolla, California, USA

**Keywords:** biomanufacturing, regenerative medicine, microgravity, International Space Station, biofabrication, stem cell therapies

## Abstract

This perspective article explores the transformative potential of biomanufacturing in low Earth orbit (LEO) for regenerative medicine. Building on key symposia and workshops, it highlights the International Space Station (ISS) National Laboratory’s role in advancing tissue engineering through microgravity research. The article discusses breakthroughs in stem cell therapies, disease modeling, and automation, while emphasizing the need for collaboration, investment, and emerging technologies like AI and machine learning. Insights from the scientific community and market analyses point to a rapidly growing sector. Strategic partnerships and policy support will be essential to scale space-based biomanufacturing and unlock new therapeutic possibilities for patients on Earth.

## Teaser

Microgravity in low Earth orbit enables breakthroughs in biomanufacturing, led by the ISS National Lab. This article highlights advances in regenerative medicine, stem cell therapies, and tissue engineering. Leveraging 3D bioprinting, nanomaterials, and AI, LEO biomanufacturing transforms disease modeling and drug development, requiring public-private collaboration to reshape personalized medicine and improve health on Earth.

## Introduction

The concept of in-space biomanufacturing, which involves producing biological materials, active pharmaceutical ingredient and related intermediates, biologics, and cellular therapeutic products in the unique space environment, is a transformative innovation with the potential to revolutionize both space exploration and the astrobiotechnology industries. This idea is rooted in the understanding that the conditions of space, particularly microgravity, can significantly alter and enhance biological processes that are challenging or less efficient under Earth’s gravity. Without significant gravitational forces, cells, tissues, and other three-dimensional (3D) biological structures as well as complex proteins and an expanding class of natural product-derived drugs with multiple stereocenters behave differently, creating opportunities for entirely new forms of manufacturing that were previously unimaginable. The origins of in-space biomanufacturing can be traced back to the early days of space exploration when scientists first observed the effects of microgravity on biological systems. Experiments conducted during space missions in the 1980s and 1990s, especially on space shuttles and the MIR Space Station, provided critical insights (Marbarger, 1998). Researchers discovered that microgravity influences cell behavior (Blaber et al., 2014; Thiel et al., 2012), leading to the hypothesis that these altered conditions could be harnessed for biomanufacturing. One of the earliest successes in this area was the growth of protein crystals in space (DeLucas et al., 1986). Unfortunately, a large portion of the research findings from these experiments have not been widely published in scientific journals and are only available in internal reports from space agencies. Research conducted by space agencies like National Aeronautics and Space Administration (NASA) is often reported in internal documents, technical reports, or mission summaries that are not widely distributed to the general scientific community or the public. In some cases, data from space-based research may be considered sensitive or strategic, limiting its open dissemination. Agencies may restrict data to collaborators or internal stakeholders. This is particularly true for experiments that resulted in failures or marginal successes, which must be considered noteworthy for publication in the broader scientific literature. In some cases, results might have reached the press, but the overall record of experiments conducted during the 1970s and 1980s still needs to be completed. On Earth, gravity often causes imperfections in crystal growth, but in microgravity, proteins can form larger and more perfectly structured crystals, which are invaluable in pharmaceutical research for enabling more precise drug design and development. The establishment of the International Space Station (ISS) in the 2000s marked a significant milestone in advancing the concept of in-space biomanufacturing. With its state-of-the-art laboratories and long-duration missions, the ISS provided a stable platform for conducting more complex and long-term biological experiments. Scientists began to explore not only protein crystallization but also the potential for tissue engineering and 3D bioprinting in space.

For instance, 3D bioprinting, a technology that allows the creation of tissue-like structures using cells and biomaterials, has shown promising results in the microgravity environment of the ISS. In space, the lack of gravity enables the formation of more intricate and stable 3D structures, which could be used for regenerative medicine, organ transplantation, and personalized medicine (Tabury et al., 2023; Van Ombergen et al., 2023). In microgravity, the absence of gravity eliminates the compressive forces that tissues experience on Earth. This allows for the creation of more open and interconnected structures, potentially leading to better vascularization and cell organization. Some studies suggest that microgravity can promote increased cell proliferation and differentiation, resulting in more mature and functional tissues (Blaber et al., 2015; Lei et al., 2018). The lack of gravity can lead to a more even distribution of cells and biomaterials within the printed construct, potentially improving tissue uniformity (van den Nieuwenhof et al., 2024). Adapting bioprinters to the microgravity environment presents significant challenges, including fluid behavior, handling of bio-inks in weightlessness, and ensuring proper deposition of cells (Tabury et al., 2023). The growing involvement of private companies in space exploration has significantly accelerated the development of in-space biomanufacturing. In collaboration with NASA and other space agencies, private companies are investing heavily in exploring the commercial potential of manufacturing biological products, accelerating protein crystallization for target identification, and developing targeted therapies in 3D tissue stem cell organoids in space. In collaboration with NASA and other space agencies, private companies are investing heavily in exploring the commercial potential of manufacturing biological products, biologics, and small-molecule targeted therapeutics in space. Biotechnology and pharmaceutical companies, for instance, are leveraging microgravity to produce high-quality protein crystals, which can lead to more precise and effective drug formulations (Reichert et al., 2019). Similarly, the unique conditions of microgravity have been shown to enhance the growth of tissue organoids, stem cells, and cancer, opening new avenues for regenerative medicine and cancer breakthroughs (Ghani and Zubair, 2024). Other efforts focus on developing advanced biomaterials, such as 3D-printed tissues and scaffolds, which could revolutionize medical treatments and transplantation technologies (Bishop et al., 2017).

The prospect of manufacturing these high-value products in space has sparked significant interest from the biotech industry. These innovations hold the potential to not only support long-duration space missions but also address critical medical and scientific challenges on Earth, offering transformative solutions for healthcare. In addition to commercial interests, in-space biomanufacturing is also driven by the practical needs of long-term space exploration. Missions to Mars and other deep-space destinations require sustainable solutions for food, medicine, and other essential supplies. In-space biomanufacturing could enable the production of these necessities on-site, reducing the need for resupply missions from Earth and making long-duration missions more feasible. For example, the ability to bioprint tissues or even organs in space could be crucial for treating injuries or illnesses during extended missions. While this represents a compelling long-term goal, and the potential of in-space biomanufacturing is vast, significant challenges remain. The technology for large-scale biomanufacturing in space is still in its infancy, and many technical and logistical hurdles must be addressed. These include developing reliable and efficient bioreactors that can operate in microgravity, addressing the challenges of transporting and maintaining biological materials in space and ensuring the safety and efficacy of space-manufactured products. To our knowledge, no peer-reviewed studies to date have comprehensively modeled the full resource and infrastructure requirements for in-flight organ bioprinting and implantation. This concept, while strategically valuable, should be viewed as a horizon vision that will require decades of technological and clinical maturation before implementation is feasible in operational spaceflight scenarios. However, progress has been made through cutting-edge cryopreservation techniques and thermal insulation technologies, such as the cryogenic freezers on the ISS, which enable long-term preservation of biological samples. The Minus Eighty-degree Laboratory Freezer for the ISS, with its four insulated dewars that can be independently set to +2°C, −35°C, or −95°C, plays a crucial role in this preservation. Additionally, CO_2_-independent cell culture methods have simplified the transportation and cultivation of cells in space by eliminating the need for CO_2_-dependent devices (Preininger et al., 2016). Automated bioreactors, along with improved radiation shielding, further stabilize cell growth conditions. Notably, advances in medium formulation and stability would reduce the need for frequent medium exchanges and minimize cold storage requirements, significantly lowering biomanufacturing costs. A major breakthrough has been the application of static culture systems, originally developed for the pioneering work aboard the ISS using patient-derived brain cell 3D organoid models for neurodegeneration to reduce media exchanges (Marotta et al., 2024), which has been successfully extended to patient-derived tumor organoids. These technological innovations ensure the viability of biological materials in space, supporting long-term research and biomanufacturing in low Earth orbit (LEO). However, ethical considerations surrounding the manipulation of biological systems in space and the potential environmental impact of such activities must also be addressed to ensure responsible scientific progress.

Cryopreservation is a valid and valuable technology in biomanufacturing for long-term storage of biological materials (Jaiswal and Vagga, 2022; Preininger et al., 2016). However, in LEO, the full implementation of cryopreservation is constrained by limited access to the necessary enabling technologies (Aijaz et al., 2018). The unique challenges of operating in microgravity require a more pragmatic approach to biological sample management. As a result, fresh sample return is prioritized. It offers more immediate and effective solutions for downstream analyses once back on Earth. Moreover, space-based biomedical research faces multiple logistical concerns that extend beyond cryopreservation, including those related to biomanufacturing processes, quality control procedures, and the distribution of biological products to patients. We recognize that these logistical challenges are further compounded by the off-Earth location of our facilities, where transportation and storage face more significant limitations than terrestrial counterparts. Incorporating these considerations into our program, we aim to optimize the management and preservation of biological materials under the unique constraints of space-based operations, ensuring both the integrity of our processes and the reliability of our outcomes in biomanufacturing and biological product distribution.

Despite these challenges, the future of in-space biomanufacturing is promising. As technology advances and our understanding of microgravity’s effects on biological processes deepens, the possibilities for in-space biomanufacturing will continue to expand. This field could play a crucial role in supporting human life in space while driving innovation in biotechnology, medicine, and materials science on Earth. The ongoing collaboration between public space agencies, private companies, and research institutions will be key to realizing the full potential of in-space biomanufacturing, making it a cornerstone of future space exploration and a significant contributor to human health and well-being.

The burgeoning field of biomanufacturing in LEO represents a frontier with unprecedented potential for regenerative medicine. Research facilitated by the ISS National Lab over the past decade has shown how the unique microgravity conditions of LEO can significantly advance our understanding and capabilities in tissue engineering and regenerative medicine. The distinct microgravity environment in LEO presents conditions for cellular behavior, tissue development, and biomanufacturing processes that are difficult to fully replicate on Earth. While ground-based platforms such as clinostats, rotating wall vessels, and random positioning machines can simulate the aspects of microgravity and have proven valuable in preliminary research and hypothesis generation, they introduce mechanical shear forces and rotational artifacts that can affect cellular responses. These systems are essential for experimental optimization and cost-effective preparatory studies, but they do not fully recapitulate the near weightlessness of LEO, where gravitational vectors are effectively nullified. Therefore, validation of key findings under real microgravity conditions aboard the ISS remains indispensable for ensuring translational relevance and robust biomanufacturing outcomes in space-based biomedical research. The physiological changes experienced by astronauts in microgravity—such as cardiovascular deconditioning, muscle atrophy, bone loss, and stem cell and immune dysfunction—mimic the effects of aging and chronic diseases, providing opportunities for valuable research. This parallel allows for the exploration of disease progression and therapeutic testing within time frames that are not possible under terrestrial conditions. Additionally, the microgravity environment facilitates the bio-fabrication of complex biological structures (Moroni et al., 2022), the crystallization of complex molecules, and the novel formulation of therapeutics (Reichert et al., 2019), offering advantages in using lower viscosity bio-inks, creating new solutions for tissue engineering. Recent initiatives have expanded the application of microgravity to include the development of stem cell therapies (Arzt et al., 2024; Baio et al., 2018; Ghani and Zubair, 2024; Huang et al., 2020; Hwang et al., 2023), disease modeling (Jogdand et al., 2024; Low and Giulianotti, 2020), and the biofabrication of tissues and organs (Moroni et al., 2022; Sharma et al., 2022; Van Ombergen et al., 2023). These advancements promise to enhance our understanding of human biology and disease mechanisms, accelerate drug development, and revolutionize the production of regenerative therapies. The collaborative efforts of the 2020 Biomanufacturing in Space Symposium (Sharma et al., 2022) highlighted the importance of leveraging LEO for biomanufacturing to stimulate financial investments and overcome technical barriers, setting the stage for a sustainable market in regenerative medicine biomanufacturing in space with substantial terrestrial utility. This perspective article summarizes critical discussions exploring opportunities in disease modeling, stem cell and stem cell-derived products, and biofabrication in microgravity. Recent technological advancements, particularly the use of 3D nanobioreactors, sensor-integrated platforms (Jogdand et al., 2024), and artificial intelligence (AI)/machine learning (ML)-driven analytics (Hunsberger et al., 2020; Sharma et al., 2022), are pivotal for scaling biomanufacturing processes in LEO. Nanobioreactors allow the long-term culture of 3D organoid systems under microgravity conditions while enabling real-time monitoring of stemness, proliferation, and response to therapeutics. For example, reporter-integrated nanobioreactors were deployed in ISS missions Axiom-2 and Axiom-3 to track cancer stem cell activity and assess drug efficacy *in situ* (Crews et al., 2023; Grimm et al., 2018). Additionally, integrating biosensors into these platforms enables remote telemetry, temperature control, and metabolic readouts critical for closed-loop automation. AI and machine learning algorithms are increasingly used to optimize biomanufacturing processes through automated image analysis, experiment scheduling, and adaptive control systems (Hunsberger et al., 2020; Sharma et al., 2022). These tools can reduce astronaut workload and enable autonomous in-orbit decision-making essential for long-duration missions where crew time is limited. As interest grows in commercializing LEO for cell and tissue production, embedding AI/ML into autonomous bioreactors will be crucial to ensuring robustness, reproducibility, and quality assurance under constrained environmental and resource conditions. The ISS National Laboratory, in partnership with the National Institutes of Health (NIH), has been actively supporting the Tissue Chips in Space program, which investigates organ-on-chip systems to simulate human physiology for medical research. Publications have demonstrated the successful use of tissue chips in microgravity aboard the ISS. These studies highlight the unique advantages of microgravity for modeling human diseases and advancing tissue engineering. The studies conducted during this initiative emphasize that microgravity acts as an “accelerator” offering a novel platform for studying disease mechanisms and potential therapeutic interventions (Low and Giulianotti, 2020). In addition, Tissue Chips in Space program showed how microgravity enhances the formation of 3D cell aggregates, which more accurately mimic *in vivo* tissue architecture compared to traditional two-dimensional cultures. This advancement is crucial for improving the physiological relevance of *in vitro* models used in drug discovery and disease modeling (Jogdand et al., 2024). Additionally, it presents a market analysis projecting the growth and potential of biomanufacturing in space, as well as potential future groundbreaking discoveries in regenerative medicine (Hirschberg et al., 2022). The success of these endeavors hinges on strategic planning and policy engagement, underlining the importance of collaborative efforts in this emerging field.

## Leveraging microgravity for cancer research

The exploration of tumor organoid proliferation in 3D nanobioreactors with biosensing reporters of cancer stem cell activity in LEO represents a transformative shift in our approach to understanding and treating one of humanity’s most challenging diseases—cancer (Grimm et al., 2022). This innovative research avenue leverages the unique aspects of the microgravity environment in space to gain novel insights into cancer biology and therapeutic development. Microgravity conditions in LEO offer a distinct advantage for cancer research by providing an environment that differs significantly from Earth’s gravity. This unique setting affects cellular behavior (Bradbury et al., 2020; Corydon et al., 2023; Grimm et al., 2020; Lei et al., 2018; Lin et al., 2020; Nguyen et al., 2021; Prasad et al., 2020; Topal and Zamur, 2021; Vorselen et al., 2014), including growth, morphology, and gene expression, which are critical factors in cancer development and progression (see Table 1). Cancer cells can form more relevant 3D structures in microgravity in addition to activating retrotransposons, base deaminases, and inflammatory cytokines that promote acquisition of stem cell properties, including malignant regeneration, survival, immune evasion, and metastatic potential (Grimm et al., 2020). Microgravity has been shown to induce the formation of multicellular spheroids and organoids, providing valuable models for preclinical drug targeting and studying cancer progression at the molecular level (Grimm et al., 2022). A recent study has also shown that tumor organoids activate enzymes in LEO, like APOBEC3 and ADAR1, which are normally overexpressed during embryogenesis, albeit without the usual inhibitory signals that would preclude continuous activation terrestrially (*unpublished data*). Importantly, sustained activation of APOBEC3 and ADAR1 enzymes leads to DNA mutations and RNA alterations that enhance cancer propagation and immune evasion. Tumor organoids can provide a more rapid model for studying malignant regenerative mechanisms and have already been used for screening cancer stem-like cell-targeted therapeutics. As a proof of concept of the value of LEO in accelerating drug development, real-time confocal imaging of ADAR1 reporter expressing metastatic breast cancer organoid-containing nanobioreactors in Axiom 2 and 3 ISS missions together with downstream multi-omics analyses accelerated pre-clinical mechanism of action studies that led to Food and Drug Administration (FDA) investigational new drug (IND153126) approval of rebecsinib, a new small-molecule ADAR1 splicing inhibitor that will enter phase 1 clinical trials in 2025. Together with normal stem cell nanobioreactor therapeutic index studies, tumor organoid models in LEO will accelerate the development of more effective treatments with fewer side effects. Indeed, NASA-funded missions to the ISS have already demonstrated the superiority of one small molecular ADAR1 inhibitor, rebecsinib, over another, fedratinib, in reducing breast tumor organoid proliferation (*unpublished data*). Additional experiments in LEO will help to determine if this “cancer kill switch” is tumor specific or has more pleiotropic effects (Crews et al., 2023; Jamieson and Weissman, 2023; Pham et al., 2024; van der Werf et al., 2023).

**Table 1:** Biomanufacturing in low Earth orbit: Redefining regenerative medicine

Cell growth and morphology		
Altered cell shape and structure	in microgravity, cells adopt a more spherical shape compared with their flattened shape under normal gravity. This is because, in the absence of gravity, the forces that usually pull cells downward and cause them to spread out are absent	Tran et al. (2024)https://ntrs.nasa.gov/citations/20000085929?utm_source=chatgpt.com
Changes in cytoskeleton organization	the cytoskeleton, which provides structure to the cell, is highly sensitive to gravitational forces. In microgravity, the organization of the cytoskeleton can become disordered, affecting cell shape, division, and movement	Wu et al. (2022) Janmaleki et al. (2016)
Gene expression		
Altered gene expression	microgravity can cause changes in the expression of genes involved in various cellular processes. These changes can lead to differences in how cells behave, grow, and respond to their environment.	Sahana et al. (2023) Corydon et al. (2023)
Epigenetic changes	the microgravity environment also influences epigenetic modifications that can have long-lasting effects on cell function	Singh et al. (2010) Gambacurta et al. (2019)
Cell proliferation and differentiation		
Impact on cell division	the rate at which cells divide can be altered in microgravity. Some studies have shown that microgravity can slow down cell proliferation, while others indicate an increase in cell division, depending on the cell type	Lei et al. (2018) Wang et al. (2019)
Differentiation of stem cells	Microgravity has been found to influence the differentiation of stem cells into specialized cell types. For example, stem cells may differentiate more easily into bone, cartilage, or fat cells in microgravity, which has implications for tissue engineering and regenerative medicine	Ulbrich et al. (2014) Grimm et al. (2020)
Cell communication and signaling		
Altered intercellular communication	cells communicate with each other through signaling molecules, and microgravity can affect this communication process. The distribution and function of receptors on the cell surface, as well as the release of signaling molecules, may be altered, leading to changes in how cells interact and function together	Ullrich et al. (2008) Bradbury et al. (2020)
Changes in signal transduction pathways	microgravity can disrupt the normal pathways through which cells transmit signals, leading to changes in cellular behavior, such as how cells respond to external stimuli like growth factors or stress signals	Ullrich et al. (2008) Tauber et al. (2013)
Cell adhesion and migration		
Reduced cell adhesion	cells often show reduced adhesion to each other and to their surrounding matrix in microgravity. This is partly due to changes in the expression of adhesion molecules, which can affect the formation of tissues and the integrity of cellular structures	Bradbury et al. (2020) Bauer et al. (2020)
Altered cell migration	microgravity can influence the way cells move, often resulting in altered or slower migration. This can impact wound healing, tissue formation, and cancer metastasis studies	Radstake et al. (2023) Lin et al. (2020)
Oxidative stress and apoptosis		
Increased oxidative stress	cells in microgravity may experience increased oxidative stress, which can lead to damage in cellular components such as DNA, proteins, and lipids	Nguyen et al. (2021) Singh et al. (2021)
Apoptosis (programmed cell death)	the rate of apoptosis can be influenced by microgravity, potentially increasing in some cells due to the stress of the space environment	Prasad et al. (2020)

One of the most promising aspects of organoid production in space is its potential to accelerate drug discovery and improve the efficacy of cancer treatments. Traditional 2D cell culture methods on Earth fail to replicate the complex 3D interactions among cancer cells and their microenvironment, which can significantly impact drug response. By facilitating the growth of organoids in microgravity, researchers can better assess how cancer cells respond to therapeutic agents, allowing for the identification of more effective drug candidates and combinations. This could lead to the development of personalized medicine strategies that target specific cancer types with higher precision. Achieving success in the use of organoids on LEO-based platforms for cancer research necessitates overcoming logistical and technical challenges. It requires the development of specialized platforms and technologies designed for the unique conditions of space. Automation; flexible, gas-permeable and transparent 3D nanobioreactors for microscopic imaging in orbit; lentiviral fluorescent reporters for remote monitoring; and heated microscope stages with incubators or automated incubators with automated imaging capacity are among the innovations being developed and tested. These technologies must ensure the maintenance of optimal conditions for organoid growth, including nutrient supply, waste removal, and temperature control while enabling detailed analysis of cellular responses to treatments. The pursuit of organoid platforms for space-based cancer research underscores the importance of collaboration and investment. Space industry research institutions and biotechnology and pharmaceutical companies must work together to share knowledge, resources, and funding. Success in this field promises to attract significant industry investment and interest that will catalyze a new era of cancer research and treatments. By pooling expertise and leveraging the capabilities of the ISS and future commercial space stations, the global community can make strides toward the White House Cancer Moonshot goals to “end cancer as we know it,” and specifically to prevent more than 4 million cancer deaths by 2047 and to improve the experience of people who are touched by cancer (https://www.whitehouse.gov/cancermoonshot/).

## Leveraging microgravity for cancer research

The use of microgravity environments for 3D cell culture offers unique advantages for drug testing and therapeutic development. While it is true that cells respond differently under near-weightlessness conditions compared to Earth’s 1g environment, these differences can be exploited to reveal biological phenomena that are otherwise masked by gravitational forces. In microgravity, cells assemble into 3D structures more efficiently and uniformly without the need for scaffolds commonly used in Earth-based systems. This self-organization results in constructs that more closely mimic the architecture and function of native tissues or tumors. Studies have shown that microgravity-grown spheroids exhibit improved differentiation, polarization, and cell-cell interactions (Ma et al., 2023; Mu et al., 2022) enhancing physiological relevance of 3D constructs. Microgravity induces unique stress responses, epigenetic shifts, and protein dynamics not seen under terrestrial conditions. These altered cellular states can unveil disease-relevant molecular pathways and therapeutic targets (Aunins et al., 2018) revealing novel drug targets and mechanisms. Certain pathologies, including metastatic cancers, behave differently in microgravity, often exhibiting amplified tumorigenic properties. These enhanced phenotypes allow researchers to study disease mechanisms and progression in ways that reflect human pathophysiology more accurately, enabling the discovery of new biomarkers and therapeutic windows. In addition, drug screening in microgravity can identify compounds with enhanced efficacy or safety under mechanical unloading and other stress conditions. These findings are particularly relevant for vulnerable populations on Earth, such as the elderly or immobilized patients (Baran et al., 2022). This concept highlights how microgravity can help uncover therapies with broader physiological relevance. To address the concern regarding applicability to Earth-based medicine, microgravity studies serve as a critical complement to terrestrial research. They validate hypotheses and expose hidden biological mechanisms, thereby improving the predictive value of *in vitro* models and drug testing pipelines. Importantly, we are now collecting results—some not yet published—that reveal cellular behaviors and therapeutic responses unachievable in standard Earth-based systems. These insights are accelerating our understanding of cellular biology and informing the development of next-generation therapeutics.

## Emerging technologies in space biomanufacturing

The integration of innovative technologies—such as DNA-inspired nanomaterials, advanced 3D printing techniques, and stem cell and tissue production methods—presents an unprecedented opportunity to enhance our capabilities beyond Earth’s confines. This expansion holds promise for groundbreaking advancements in various scientific fields and exemplifies the necessity of a united, collaborative effort to reach new heights of innovation.

### DNA-inspired nanomaterials

Utilizing the intrinsic properties of DNA to design and synthesize nanomaterials could lead to the development of highly specific and functional materials. The biomimetic self-assembly capabilities of Janus base materials enable the creation of targeted drug delivery systems with enhanced uniformity and drug/gene loading efficiency, offering new avenues for delivery that also provide advantages in distribution logistics due to their stability at room temperature and reduced toxicity over current lipid nanoparticle delivery systems. Additionally, developing first-in-kind injectable scaffolds using these nanomaterials could revolutionize regenerative medicine, facilitating the repair and regeneration of tissues and organs for patients on Earth.

## Acceleration of small-molecule, biologic, and cellular therapeutics development in space

Targeted protein crystallization with small-molecule and monoclonal antibody inhibitors has accelerated as a result of the successful use of microgravity to nucleate more homogeneous protein crystals bound to small-molecule drugs and monoclonal antibodies. This was exemplified by the protein crystallization of Keytruda, a monoclonal antibody binding to its target programmed cell death protein 1 in LEO, thereby leading to a change to an injectable formulation that is more widely available to patients. Moreover, flow chemistry in microgravity may be used efficiently to enhance the production of intermediates of natural product-derived small-molecule drugs with multiple stereocenters (chiral centers) where ring-closing metathesis is often constrained by gravity. An example of this is rebecsinib, a multiple chiral center small molecule that inhibits ADAR1, a driver of malignant regeneration and immune evasion in over 20 cancers. Finally, cellular therapeutics, including chimeric antigen receptor T cells, may be expanded more efficiently in microgravity, which induces cell proliferation.

### 3D bioprinting

Adapting 3D printing technology for space use could revolutionize how we think about biomanufacturing on Earth and beyond. The microgravity conditions in LEO offer a conducive environment for 3D bioprinting, allowing for the fabrication of 3D scaffold-free complex tissue structures (Rezapour Sarabi et al., 2023). Without the constraints of gravity, bioprinted tissues can be produced more uniformly, with unique geometries that exhibit improved cell organization, providing potential advantages for regenerative medicine applications on Earth. Additionally, insights gained from studying tissue development in microgravity could inform the design of more effective bioprinting techniques and bio-inks for terrestrial applications, which remain challenging in terms of creating whole functional organs at a research level, and significant challenges with commercial production at scale. At the same time, microgravity offers unique advantages for bioprinting experiments, and the possibility of new methods that will address terrestrial challenges that currently limit the field, challenges such as cost, and logistics must be addressed to realize the full potential of space-based bioprinting research. Collaborative efforts among international space agencies, research institutions, and industry partners are essential to overcome these challenges and harness the transformative potential of 3D bioprinting in LEO for terrestrial benefits.

### Stem cells and tissue production in LEO

Biomanufacturing in LEO is a pioneering endeavor that harnesses the unique microgravity environment to offer significant advantages in stem cell and tissue production (Sharma et al., 2022). This transformative potential holds the promise of revolutionizing healthcare and biotechnology. Microgravity enhances the behavior of stem cells, leading to increased proliferation and differentiation (Baio et al., 2018; Huang et al., 2020; Hwang et al., 2023; Rampoldi et al., 2022). Microgravity allows cells to grow in three dimensions without scaffolds, resulting in more physiologically relevant tissue structures. It also reduces shear stress, minimizing damage to delicate cells and improving overall yield and quality (Chen et al., 2017; Grimm et al., 2020; Marotta et al., 2023). To cultivate stem cells in space, specialized bioreactors are employed to create controlled optimized environments promoting the growth of stem cells. Microgravity also fosters the formation of organoids and spheroids—3D cell cultures that mimic organ structures and functions, ideal for drug testing and disease modeling. Genetic engineering and chemical modulation further guide stem cell differentiation in LEO, enabling the production of specific cell types for various applications. While specific cell types can certainly be produced under Earth’s gravity (1g), the microgravity environment offers distinct advantages that enhance differentiation efficiency and enable novel tissue formation pathways. Microgravity minimizes shear stress, eliminates sedimentation, and supports scaffold-free 3D growth, fostering more uniform and physiologically relevant constructs. These conditions have been shown to promote enhanced lineage-specific differentiation, such as osteogenic, adipogenic, and chondrogenic fates, while also revealing unique molecular and cellular mechanisms not observed in terrestrial settings (Grimm et al., 2018). Thus, while Earth-based platforms remain essential, the unique properties of microgravity provide complementary opportunities for advancing stem cell biomanufacturing and regenerative medicine (Grimm et al., 2018). Tissue production in LEO benefits from the ability to grow more complex and organized structures compared with Earth-based methods (Grimm et al., 2018). Advanced 3D bioprinting in space enables the creation of tissue constructs layer by layer, resulting in highly organized and functional tissues. Additionally, while microgravity lessens the need for scaffolds, hydrogels can be used to support tissue growth initially. The potential applications of these advancements are vast and exciting. In regenerative medicine, producing tissues and organs for transplantation becomes more feasible. Pharmaceutical testing benefits from more accurate models, reducing the need for animal testing and improving relevance to human health.

Enhanced disease modeling provides better insights into disease progression and treatment strategies, opening new avenues for research and development in biotechnology, regenerative medicine, and space exploration. However, space-based biomanufacturing faces challenges, such as the logistics of transporting materials and maintaining suitable conditions for cell and tissue growth in space. While advancements in space travel technology are expected to reduce the high costs associated with space missions, these costs remain significant. Public-private sector collaborations and economies of scale contribute to more frequent and affordable launches, enhancing accessibility, scalability, and the economic viability of producing high-value biomedical products in space. As launch costs continue to decline, the potential for space-based biomanufacturing to revolutionize healthcare and biotechnology becomes increasingly attainable, fostering innovation and accelerating product development. However, it is important to remember that addressing regulatory and ethical issues is not just a task but a responsibility. It is crucial for ensuring compliance and managing concerns related to space-based biomanufacturing. By being aware of these challenges, we can better prepare for and navigate the complexities of this innovative field and demonstrate our commitment to the cause.

It is true that cells grown under microgravity (μg) conditions exhibit significant differences in gene expression, protein profiles, and functional behavior compared to cells grown in a 1g environment. When considering their use in cell therapies, the potential for these cells to reconfigure or adapt to Earth’s gravity must be carefully examined. Recent studies have shown that microgravity-induced changes in cells, such as epigenetic modifications and alterations in transcriptional profiles, are often maintained even after the cells are returned to 1g environments, albeit to varying degrees. For example, it has been demonstrated that certain stress-response genes remained upregulated in microgravity-grown cells upon their reintroduction to 1g, indicating a degree of stability in their reprogrammed state. This epigenetic memory can be leveraged for therapeutic applications where specific traits, such as enhanced regenerative capacity or reduced inflammatory profiles, are desirable (Beheshti et al., 2021). Microgravity can also act as a preconditioning environment that primes cells for therapeutic use. For instance, mesenchymal stem cells cultured in microgravity have shown enhanced proliferative capacity, improved immunomodulatory effects, and reduced senescence compared to their Earth-grown counterparts (Grimm et al., 2020). These enhanced traits could potentially improve therapeutic outcomes, even if partial reconfiguration occurs upon reintegration into a 1g environment. While some microgravity-induced changes are transient and revert upon exposure to 1g, others are more sustained and functional. For example, studies on cardiomyocytes and neural stem cells suggest that structural and biochemical adaptations, such as improved differentiation potential or resistance to oxidative stress, persist long enough to make these cells highly valuable for short-term therapeutic applications, such as tissue repair following injury or ischemia (Rampoldi et al., 2022). The use of microgravity-grown cells may involve optimizing the therapeutic window, such that cells are delivered and utilized before significant reconfiguration occurs. This could include encapsulating or stabilizing the cells in a microenvironment that helps maintain their desired properties during the transition to 1g. Before deployment in cell therapies, it is essential to validate that microgravity-induced traits translate into measurable therapeutic benefits under 1g conditions. Co-culture experiments, organ-on-chip models, and animal studies in terrestrial environments provide crucial preclinical data to evaluate their efficacy and stability. Biomanufacturing in microgravity is not just about producing cells but also about understanding and harnessing the unique changes induced by this environment. For example, microgravity can be used to optimize cell expansion or differentiation protocols, which are then finalized and standardized for Earth-based clinical use. There are several practical considerations for translating microgravity-grown cells into clinical applications. One important factor is the therapeutic window; space-grown cells may need to be delivered shortly after return to Earth to maximize the benefits of microgravity-induced traits or, alternatively, stabilized within biomaterials that help preserve these desirable properties during the transition to a 1g environment. Before clinical deployment, it is essential to validate that microgravity-induced phenotypes translate into functional therapeutic advantages under Earth conditions. This requires rigorous *in vitro* testing through co-culture systems, organ-on-chip platforms, and *in vivo* animal models to ensure safety, efficacy, and reproducibility. Moreover, microgravity serves not only as a manufacturing environment but also as a unique discovery platform for identifying novel cell conditioning and differentiation protocols. These insights can inform the development of standardized, Earth-compatible workflows that incorporate the advantages of microgravity into regenerative medicine and cellular therapy pipelines.

## Automation, robotics, and the human element

Technological advancements and human expertise play crucial roles in space biomanufacturing. As we stand on the brink of expanding our presence in space, integrating automation and robotics emerges as a critical factor in enhancing operational efficiency and scalability. However, this technological evolution does not diminish the vital importance of the human element. Instead, it prompts a reevaluation of how humans and machines can best collaborate to achieve the ambitious space exploration and commercialization goals.

Adopting automation and robotics in space biomanufacturing represents a significant leap toward higher efficiency, precision, and scalability. Automated systems can perform repetitive and precise tasks, manage complex data collection and analysis, and operate continuously without the constraints of human fatigue. Despite the strides in automation and robotics, the human element remains irreplaceable. Human judgment, adaptability, and creativity are essential, especially when unexpected situations arise. Astronauts and technicians bring problem-solving and decision-making capabilities that, at least for now, machines cannot fully replicate. Their ability to interact intuitively with the environment, tools, and systems provides a layer of versatility and innovation. Furthermore, human presence in space missions adds intrinsic value to exploration. It inspires, brings perspective, and connects humanity. As such, the role of humans in space-based biomanufacturing processes is also evolving from hands-on operational tasks to more strategic oversight, monitoring, and intervention roles, where crew members guide and collaborate with automated systems to achieve mission objectives.

Finding the optimal balance among automation, robotics, and human involvement is dependent on the stage of product development. While in the early stages, human interaction with semi-automated processes is critical. Once a process is locked, the need and desire for human intervention, from a regulatory perspective, decreases, as it reduces human error and increases process control. This balance involves assessing the trade-offs between the cost and logistics of human presence in space against the efficiency and capabilities of automated systems. The design and planning of missions must consider these factors alongside the safety and well-being of human crew members. As space biomedical missions become more complex and the space economy grows, the integration of humans and machines must be dynamic and adaptable. Training programs for astronauts and ground control personnel will evolve to ensure they can effectively interact with and manage automated systems. Likewise, the design of robots and automated systems will increasingly need to consider human factors engineering to facilitate seamless human-machine interfaces.

## Prospects and strategic planning

The transition of space-based biomanufacturing from research to product development and distribution necessitates strategic planning, prioritization, and a collaborative effort across various sectors. This process involves technological innovation and a comprehensive understanding of legal, regulatory, and economic frameworks. Strategic planning is crucial, as it focuses on projects with significant potential for societal benefit and requires a multidisciplinary approach to adapt and scale discoveries to commercial production. Collaboration with policymakers is essential for creating a supportive environment, establishing clear intellectual property pipelines to protect innovations. Assessing economic viability, understanding regulatory paths for products intended for human use, and adopting a product-specific approach are key to addressing the unique challenges of space biomanufacturing. The future success of this endeavor will depend on innovative thinking; partnerships among governments, industries, and academia; and the space community’s ability to leverage the unique advantages of the space environment.

As we embark on this new era, the potential for biomanufacturing in space holds the promise of extending the boundaries of science and technology as we know them, offering opportunities for groundbreaking advancements that could benefit humanity in a way that will create a paradigm shift of technology and healthcare. Microgravity catalyzes regenerative medicine, offering promise for advancing tissue engineering, disease modeling, and the bio-fabrication of tissues and organs. These conditions, unattainable on Earth, hold the potential to revolutionize our understanding and treatment of various diseases, ushering in a new era of medical innovation. The indispensable role of automation, AI, and ML in developing efficient and scalable biomanufacturing processes in space is underscored. These transformative technologies promise to automate experimental runs, minimize astronaut intervention, and unlock novel discoveries through advanced data analysis.

The recurring theme of collaboration among space agencies, research institutions, and biotechnology and pharmaceutical companies is emphasized as pivotal for overcoming technical barriers, stimulating financial investment, and establishing a sustainable market for space-based biomanufacturing. A comprehensive market analysis suggests significant growth potential for biomanufacturing in space, positioning it as a critical driver of advancements in regenerative medicine (Hirschberg et al., 2022). However, this perspective article also highlights the necessity for strategic planning and engagement with policymakers and regulatory authorities to navigate the unique challenges of space biomanufacturing effectively. Clear intellectual property guidelines are essential to protect innovations and foster a conducive environment for investment and innovation. Addressing intellectual property rights and legal and regulatory aspects in space presents a complex challenge, requiring international collaboration and deliberation over jurisdiction, innovation protection, and equitable sharing of space-derived benefits. Earthbound regulatory authorities often have programs designed to provide early feedback on regulatory science and policy challenges for advanced manufacturing such as space biomanufacturing. An example is the FDA Center for Biologics Evaluation and Research (CBER) Advanced Technologies Team, which conducts informational meetings to introduce FDA staff to innovative manufacturing technology. Indeed, early discussions have been conducted with the FDA CBER Advanced Technologies Team, and continuing dialogue is planned.

## Conclusion

Here, we present a forward-looking vision for the future of biomanufacturing in space, highlighting its transformative potential to advance regenerative medicine. While foundational research remains critical, recent breakthroughs have already paved the way for translational progress: for example, the ADAR1 inhibitor rebecsinib, developed and tested through organoid models in microgravity, received FDA IND clearance (IND153126) and is expected to enter phase 1 clinical trials in 2025. Several other programs, such as stem cell-based therapeutics investigated on the ISS under the NIH-funded Tissue Chips in Space program, have laid the groundwork for clinical translation, with preclinical results submitted or being prepared for regulatory review. Registered trials and translational applications are beginning to emerge (e.g., NCT06099964). Thus, space-based platforms are transitioning from fundamental science to translational and clinical impact. Realizing this future will still require navigating technological, regulatory, and ethical complexities, but the convergence of microgravity research, automation, and biomanufacturing is driving the field steadily forward. However, realizing this vision will necessitate navigating a complex landscape of technological, legal, ethical, regulatory, and environmental challenges, which are expected to remain central to ongoing discussions and debates. Collaboration, innovation, and careful proactive consideration of these challenges will be essential in shaping the future of space-based biomanufacturing and its impact on benefits to humanity for future medical breakthroughs and continued exploration beyond Earth.

## Declaration of interests

The authors declare that they have a potential competing interest related to the subject matter of this publication. Specifically, a patent application has been filed with the United States Patent and Trademark Office under US patent application no. 18/700,180, which pertains to aspects of the research and findings discussed in this paper. The patent application covers the new method of culturing brain organoid in microgravity using static systems. In addition, the small-molecule inhibitor of ADAR1 splicing, rebecsinib, is owned by Aspera Biomedicines co-founded by C.H.M.J.

## References

[bib1] Aijaz A., Li M., Smith D., Khong D., LeBlon C., Fenton O.S., Olabisi R.M., Libutti S., Tischfield J., Maus M.V. (2018). Biomanufacturing for clinically advanced cell therapies. Nat. Biomed. Eng..

[bib2] Arzt M., Mozneb M., Escopete S., Moses J., Sharma A. (2024). The Benefits of Stem Cell Biology and Tissue Engineering in Low-Earth Orbit. Stem Cell. Dev..

[bib3] Aunins T.R., Erickson K.E., Prasad N., Levy S.E., Jones A., Shrestha S., Mastracchio R., Stodieck L., Klaus D., Zea L., Chatterjee A. (2018). Spaceflight Modifies Escherichia coli Gene Expression in Response to Antibiotic Exposure and Reveals Role of Oxidative Stress Response. Front. Microbiol..

[bib4] Baio J., Martinez A.F., Bailey L., Hasaniya N., Pecaut M.J., Kearns-Jonker M. (2018). Spaceflight Activates Protein Kinase C Alpha Signaling and Modifies the Developmental Stage of Human Neonatal Cardiovascular Progenitor Cells. Stem Cell. Dev..

[bib5] Baran R., Wehland M., Schulz H., Heer M., Infanger M., Grimm D. (2022). Microgravity-Related Changes in Bone Density and Treatment Options: A Systematic Review. Int. J. Mol. Sci..

[bib6] Bauer T.J., Gombocz E., Wehland M., Bauer J., Infanger M., Grimm D. (2020). Insight in Adhesion Protein Sialylation and Microgravity Dependent Cell Adhesion-An Omics Network Approach. Int. J. Mol. Sci..

[bib7] Beheshti A., McDonald J.T., Hada M., Takahashi A., Mason C.E., Mognato M. (2021). Genomic Changes Driven by Radiation-Induced DNA Damage and Microgravity in Human Cells. Int. J. Mol. Sci..

[bib8] Bishop E.S., Mostafa S., Pakvasa M., Luu H.H., Lee M.J., Wolf J.M., Ameer G.A., He T.-C., Reid R.R. (2017). 3-D bioprinting technologies in tissue engineering and regenerative medicine: Current and future trends. Genes Dis..

[bib9] Blaber E.A., Dvorochkin N., Torres M.L., Yousuf R., Burns B.P., Globus R.K., Almeida E.a.C. (2014). Mechanical unloading of bone in microgravity reduces mesenchymal and hematopoietic stem cell-mediated tissue regeneration. Stem Cell Res..

[bib10] Blaber E.A., Finkelstein H., Dvorochkin N., Sato K.Y., Yousuf R., Burns B.P., Globus R.K., Almeida E.A.C. (2015). Microgravity Reduces the Differentiation and Regenerative Potential of Embryonic Stem Cells. Stem Cell. Dev..

[bib11] Bradbury P., Wu H., Choi J.U., Rowan A.E., Zhang H., Poole K., Lauko J., Chou J. (2020). Modeling the Impact of Microgravity at the Cellular Level: Implications for Human Disease. Front. Cell Dev. Biol..

[bib12] Chen Z., Luo Q., Yuan L., Song G. (2017). Microgravity directs stem cell differentiation. Histol. Histopathol..

[bib13] Corydon T.J., Schulz H., Richter P., Strauch S.M., Böhmer M., Ricciardi D.A., Wehland M., Krüger M., Erzinger G.S., Lebert M. (2023). Current Knowledge about the Impact of Microgravity on Gene Regulation. Cells.

[bib14] Crews L.A., Ma W., Ladel L., Pham J., Balaian L., Steel S.K., Mondala P.K., Diep R.H., Wu C.N., Mason C.N. (2023). Reversal of malignant ADAR1 splice isoform switching with Rebecsinib. Cell Stem Cell.

[bib15] DeLucas L.J., Suddath F.L., Snyder R., Naumann R., Broom M.B., Pusey M., Yost V., Herren B., Carter D., Nelson B. (1986). Preliminary investigations of protein crystal growth using the space shuttle. J. Cryst. Growth.

[bib16] Gambacurta A., Merlini G., Ruggiero C., Diedenhofen G., Battista N., Bari M., Balsamo M., Piccirillo S., Valentini G., Mascetti G., Maccarrone M. (2019). Human osteogenic differentiation in Space: proteomic and epigenetic clues to better understand osteoporosis. Sci. Rep..

[bib17] Ghani F., Zubair A.C. (2024). Discoveries from human stem cell research in space that are relevant to advancing cellular therapies on Earth. NPJ Microgravity.

[bib18] Grimm D., Wehland M., Corydon T.J., Richter P., Prasad B., Bauer J., Egli M., Kopp S., Lebert M., Krüger M. (2020). The effects of microgravity on differentiation and cell growth in stem cells and cancer stem cells. Stem Cells Transl. Med..

[bib19] Grimm D., Egli M., Krüger M., Riwaldt S., Corydon T.J., Kopp S., Wehland M., Wise P., Infanger M., Mann V., Sundaresan A. (2018). Tissue Engineering Under Microgravity Conditions–Use of Stem Cells and Specialized Cells. Stem Cell. Dev..

[bib20] Grimm D., Schulz H., Krüger M., Cortés-Sánchez J.L., Egli M., Kraus A., Sahana J., Corydon T.J., Hemmersbach R., Wise P.M. (2022). The Fight against Cancer by Microgravity: The Multicellular Spheroid as a Metastasis Model. Int. J. Mol. Sci..

[bib21] Hirschberg C., Kulish I., Rozenkopf I., Sodoge T. (2022).

[bib22] Huang P., Russell A.L., Lefavor R., Durand N.C., James E., Harvey L., Zhang C., Countryman S., Stodieck L., Zubair A.C. (2020). Feasibility, potency, and safety of growing human mesenchymal stem cells in space for clinical application. NPJ Microgravity.

[bib23] Hunsberger J., Simon C., Zylberberg C., Ramamoorthy P., Tubon T., Bedi R., Gielen K., Hansen C., Fischer L., Johnson J. (2020). Improving patient outcomes with regenerative medicine: How the Regenerative Medicine Manufacturing Society plans to move the needle forward in cell manufacturing, standards, 3D bioprinting, artificial intelligence-enabled automation, education, and training. Stem Cells Transl. Med..

[bib24] Hwang H., Rampoldi A., Forghani P., Li D., Fite J., Boland G., Maher K., Xu C. (2023). Space microgravity increases expression of genes associated with proliferation and differentiation in human cardiac spheres. NPJ Microgravity.

[bib25] Jaiswal A.N., Vagga A. (2022). Cryopreservation: A Review Article. Cureus.

[bib26] Jamieson C.H.M., Weissman I.L. (2023). Stem-Cell Aging and Pathways to Precancer Evolution. N. Engl. J. Med..

[bib27] Janmaleki M., Pachenari M., Seyedpour S.M., Shahghadami R., Sanati-Nezhad A. (2016). Impact of Simulated Microgravity on Cytoskeleton and Viscoelastic Properties of Endothelial Cell. Sci. Rep..

[bib28] Jogdand A., Landolina M., Chen Y. (2024). Organs in orbit: How tissue chip technology benefits from microgravity, a perspective. Front. Lab Chip Technol..

[bib29] Lei X., Cao Y., Zhang Y., Qian J., Zhao Q., Liu F., Zhang T., Zhou J., Gu Y., Xia G., Duan E. (2018). Effect of microgravity on proliferation and differentiation of embryonic stem cells in an automated culturing system during the TZ-1 space mission. Cell Prolif..

[bib30] Lin X., Zhang K., Wei D., Tian Y., Gao Y., Chen Z., Qian A. (2020). The Impact of Spaceflight and Simulated Microgravity on Cell Adhesion. Int. J. Mol. Sci..

[bib31] Low L.A., Giulianotti M.A. (2019). Tissue Chips in Space: Modeling Human Diseases in Microgravity. Pharm. Res..

[bib32] Ma C., Duan X., Lei X. (2023). 3D cell culture model: From ground experiment to microgravity study. Front. Bioeng. Biotechnol..

[bib34] Marotta D., Ijaz L., Barbar L., Nijsure M., Stein J., Clements T., Stoudemire J., Grisanti P., Noggle S.A., Loring J.F., Fossati V. (2023). Studies on the International Space Station to assess the effects of microgravity on iPSC-derived neural organoids. bioRxiv.

[bib35] Marotta D., Ijaz L., Barbar L., Nijsure M., Stein J., Pirjanian N., Kruglikov I., Clements T., Stoudemire J., Grisanti P. (2024). Effects of microgravity on human iPSC-derived neural organoids on the International Space Station. Stem Cells Transl. Med..

[bib36] Moroni L., Tabury K., Stenuit H., Grimm D., Baatout S., Mironov V. (2022). What can biofabrication do for space and what can space do for biofabrication?. Trends Biotechnol..

[bib37] Mu X., He W., Rivera V.A.M., De Alba R.A.D., Newman D.J., Zhang Y.S. (2022). Small tissue chips with big opportunities for space medicine. Life Sci. Space Res..

[bib38] Nguyen H.P., Tran P.H., Kim K.-S., Yang S.-G. (2021). The effects of real and simulated microgravity on cellular mitochondrial function. NPJ Microgravity.

[bib39] Pham J., Isquith J., Balaian L., Ladel L., Nandi S.P., Mack K., van der Werf I., Klacking E., Ruiz A., Mays D. (2024). Accelerated Hematopoietic Stem Cell Aging in Space. bioRxiv.

[bib40] Prasad B., Grimm D., Strauch S.M., Erzinger G.S., Corydon T.J., Lebert M., Magnusson N.E., Infanger M., Richter P., Krüger M. (2020). Influence of Microgravity on Apoptosis in Cells, Tissues, and Other Systems In Vivo and In Vitro. Int. J. Mol. Sci..

[bib41] Preininger M.K., Singh M., Xu C. (2016). Cryopreservation of Human Pluripotent Stem Cell-Derived Cardiomyocytes: Strategies, Challenges, and Future Directions. Adv. Exp. Med. Biol..

[bib42] Radstake W.E., Gautam K., Miranda S., Van Rompay C., Vermeesen R., Tabury K., Verslegers M., Dowson A., Gorissen J., van Loon J.J.W.A. (2023). Gravitational effects on fibroblasts' function in relation to wound healing. NPJ Microgravity.

[bib43] Rampoldi A., Forghani P., Li D., Hwang H., Armand L.C., Fite J., Boland G., Maxwell J., Maher K., Xu C. (2022). Space microgravity improves proliferation of human iPSC-derived cardiomyocytes. Stem Cell Rep..

[bib44] Reichert P., Prosise W., Fischmann T.O., Scapin G., Narasimhan C., Spinale A., Polniak R., Yang X., Walsh E., Patel D. (2019). Pembrolizumab microgravity crystallization experimentation. NPJ Microgravity.

[bib45] Rezapour Sarabi M., Yetisen A.K., Tasoglu S. (2023). Bioprinting in Microgravity. ACS Biomater. Sci. Eng..

[bib46] Sahana J., Cortés-Sánchez J.L., Sandt V., Melnik D., Corydon T.J., Schulz H., Cai Z., Evert K., Grimm D., Wehland M. (2023). Long-Term Simulation of Microgravity Induces Changes in Gene Expression in Breast Cancer Cells. Int. J. Mol. Sci..

[bib47] Sharma A., Clemens R.A., Garcia O., Taylor D.L., Wagner N.L., Shepard K.A., Gupta A., Malany S., Grodzinsky A.J., Kearns-Jonker M. (2022). Biomanufacturing in low Earth orbit for regenerative medicine. Stem Cell Rep..

[bib48] Singh K.P., Kumari R., Dumond J.W. (2010). Simulated microgravity-induced epigenetic changes in human lymphocytes. J. Cell. Biochem..

[bib49] Singh R., Rajput M., Singh R.P. (2021). Simulated microgravity triggers DNA damage and mitochondria-mediated apoptosis through ROS generation in human promyelocytic leukemic cells. Mitochondrion.

[bib50] Tabury K., Rehnberg E., Baselet B., Baatout S., Moroni L. (2023). Bioprinting of Cardiac Tissue in Space: Where Are We?. Adv. Healthcare Mater..

[bib51] Tauber S., Hauschild S., Crescio C., Secchi C., Paulsen K., Pantaleo A., Saba A., Buttron I., Thiel C.S., Cogoli A. (2013). Signal transduction in primary human T lymphocytes in altered gravity – results of the MASER-12 suborbital space flight mission. Cell Commun. Signal..

[bib52] Thiel C.S., Paulsen K., Bradacs G., Lust K., Tauber S., Dumrese C., Hilliger A., Schoppmann K., Biskup J., Gölz N. (2012). Rapid alterations of cell cycle control proteins in human T lymphocytes in microgravity. Cell Commun. Signal..

[bib53] Topal U., Zamur C. (2021). Microgravity, Stem Cells, and Cancer: A New Hope for Cancer Treatment. Stem Cell. Int..

[bib54] Tran M.T., Ho C.N.Q., Hoang S.N., Doan C.C., Nguyen M.T., Van H.D., Ly C.N., Le C.P.M., Hoang H.N.Q., Nguyen H.T.M. (2024). Morphological Changes of 3T3 Cells under Simulated Microgravity. Cells.

[bib55] Ulbrich C., Wehland M., Pietsch J., Aleshcheva G., Wise P., van Loon J., Magnusson N., Infanger M., Grosse J., Eilles C. (2014). The impact of simulated and real microgravity on bone cells and mesenchymal stem cells. BioMed Res. Int..

[bib56] Ullrich O., Huber K., Lang K. (2008). Signal transduction in cells of the immune system in microgravity. Cell Commun. Signal..

[bib57] van den Nieuwenhof D.W.A., Moroni L., Chou J., Hinkelbein J. (2024). Cellular response in three-dimensional spheroids and tissues exposed to real and simulated microgravity: A narrative review. NPJ Microgravity.

[bib58] van der Werf I., Mondala P.K., Steel S.K., Balaian L., Ladel L., Mason C.N., Diep R.H., Pham J., Cloos J., Kaspers G.J.L. (2023). Detection and targeting of splicing deregulation in pediatric acute myeloid leukemia stem cells. Cell Rep. Med..

[bib59] Van Ombergen A., Chalupa-Gantner F., Chansoria P., Colosimo B.M., Costantini M., Domingos M., Dufour A., De Maria C., Groll J., Jungst T. (2023). 3D Bioprinting in Microgravity: Opportunities, Challenges, and Possible Applications in Space. Adv. Healthcare Mater..

[bib60] Vorselen D., Roos W.H., MacKintosh F.C., Wuite G.J.L., van Loon J.J.W.A. (2014). The role of the cytoskeleton in sensing changes in gravity by nonspecialized cells. FASEB J..

[bib61] Wang P., Tian H., Zhang J., Qian J., Li L., Shi L., Zhao Y. (2019). Spaceflight/microgravity inhibits the proliferation of hematopoietic stem cells by decreasing Kit-Ras/cAMP-CREB pathway networks as evidenced by RNA-Seq assays. FASEB J..

[bib62] Wu X.-T., Yang X., Tian R., Li Y.H., Wang C.Y., Fan Y.B., Sun L.W. (2022). Cells respond to space microgravity through cytoskeleton reorganization. FASEB J..

[bib33] Marbarger J.P. (1998). Early history of space biology and medicine. Acta Astronaut.

